# ‘In practice we don’t use that much theory’: Questioning claims of the dominance of attachment theory in children’s safeguarding social work

**DOI:** 10.1093/bjsw/bcaf068

**Published:** 2025-03-28

**Authors:** Sarah L Foster, Robbie Duschinsky

**Affiliations:** Department of Social Work, Education and Community Wellbeing, Northumbria University, Newcastle upon Tyne, NE7 7XA, UK; Department of Public Health and Primary Care, Cambridge University, Cambridge, CB2 0SZ, UK

**Keywords:** attachment, child protection, practice, social work, theory

## Abstract

There have been many calls for the use of attachment theory in children’s social work practice. Yet, some commentators claim that attachment theory has been uncritically adopted by social workers and has become an unhelpfully dominant perspective. Empirical evidence to support such claims is limited, however. We therefore sought to examine these claims in a qualitative vignette and interview study with twenty-three children’s safeguarding social workers from two English local authorities. We found that many social workers had an anti-theoretical orientation and that, for those who did draw on formal theories, attachment theory was less commonly identified as an influence than systems theory and was typically drawn on alongside other theories and perspectives. We also found examples of social workers taking a critical stance on the theory and its use in practice. The current study findings therefore challenge depictions of attachment theory as problematically dominant in children’s safeguarding social work practice. We argue that future debate on whether attachment theory has an appropriate level of practice influence would benefit from being grounded in more robust research on the current level of influence.

## Teaser

Some people argue that attachment theory is a useful perspective for children’s social workers. However, others have argued that attachment theory is not being used in helpful ways in social work practice. They are concerned it is used too much and not questioned enough. To date though, evidence on whether attachment theory is actually being overused in children’s social work practice is very limited. We therefore carried out research to investigate this further. We talked to twenty-three children’s safeguarding social workers in England and asked them to discuss a family case and tell us about whether and how they use theory in their practice. We found that many of the social workers did not use any theories in their practice. We found that those who used attachment theory tended to use it alongside other theories. We also found examples of social workers questioning how useful attachment theory is. Our findings do not therefore support claims that attachment theory is overused in children’s safeguarding social work practice. We think that it would be helpful for future debates on this topic to be better linked to research.

## Introduction

The importance of attachment theory for children’s social work has been argued many times. Multiple researchers and practitioners have recommended the use of attachment theory in child protection practice (e.g. Turney and Tanner 2001; Howe 2005; Mennen and O’Keefe 2005; O’Gorman 2013; Spieker and Crittenden 2018) and fostering and adoption (e.g. Schofield and Beek 2023). The Munro (2011) Review of Child Protection in England proposed that the minimum capabilities for child and family social work must include knowledge of attachment. A review of serious case reviews (Brandon et al. 2011) found that where there were failures to recognize problems, there was often a lack of curiosity about attachment, emotional development, and the parent–child relationship. Brandon et al. therefore argued for the need for practitioners to pay attention to these factors.

Yet running alongside calls for the use of attachment theory in children’s social work, concerns have been raised by some commentators that the theory is relied on *too much*. Smith, Cameron, and Reimer (2017), White et al. (2020), and Garrett (2023) all argue that attachment theory is an unhelpfully dominant perspective in social work practice.

Smith, Cameron, and Reimer (2017: 1606-1607) argue that, in social work practice, there is an ‘overreliance on attachment’, that attachment ‘has become the master theory to which other ways of conceiving of childcare and relationships more generally become subordinated’ and that ‘attachment’s dominance may inhibit consideration of other, complementary or alternative ideas’. White et al. (2020: 33) also claim that attachment theory is a ‘prominent, and often dominant, perspective’ in child welfare practice. Arguments about attachment theory being ‘commonplace’ and ‘dominant’ in child protection work are also repeated by Steggall and Scollen (2024: 2130). White et al. (2020: 67) propose that ideas from attachment theory have permeated into cultural discourses and norms, meaning that attachment not only operates as a formal theory but can also influence the informal theories that social workers have and thus ‘can be manifest in practice without explicit reference to the theory’.

White et al. (2020: 24) argue that attachment theory functions as an ‘undisputed fact of life’. Similarly, Garrett (2023: 2) proposes that ‘attachment thinking, particularly the work of Bowlby, forms part of the common sense of social work with children and families’. Garrett (2023: 2) also argues that, within social work, attachment operates as a ‘received idea’ and is ‘insufficiently interrogated’. He claims there is an ‘uncritical reverence’ for attachment theory proposals, which is ‘deeply problematic’ (Garrett 2023: 2, 15).

However, empirical evidence for claims that attachment theory is an uncritically accepted dominant perspective in children’s social work is limited. Smith, Cameron, and Reimer (2017) provide examples of how selective attachment ideas have been co-opted into policy, yet they do not cite or consider evidence on whether attachment theory is specifically dominant in the thinking of social workers. Garrett (2023) does not cite empirical evidence to support his claims of the dominance of this theory. Steggall and Scollen (2024) do not provide evidence of their own; instead, they cite Edwards, Gillies, and Horsley (2015) and White et al. (2020) to argue for its dominance. Yet, Edwards et al.’s research was focused on early years policy and practice rather than social work, and predominantly on the influence of brain science research rather than attachment theory. White et al. draw heavily on doctoral research with social workers by Gibson (2016), but this research does not show unequivocal support for claims of the theory’s dominance. Indeed, in his interviews with social workers about good work, Gibson found that none made explicit mention of attachment theory. Neither did Gibson observe attachment theory (or any other theory) being explicitly mentioned during 250 hours of social work team observation. Gibson did find frequent explicit references to attachment theory in the documents the social workers produced. However, the sole English local authority involved in Gibson’s research had recently received an inspection rating of inadequate, and in response social workers were required to demonstrate that their work was theory-informed. The inclusion of references to attachment theory in documents may therefore have been a localized response to this particular context, rather than evidence of the prominence of attachment theory.

The wider evidence base examining the prominence of attachment theory in practice is also limited. In a survey of 642 professionals involved in improving Children in Need’s educational outcomes in the UK (Department for Education 2018), attachment theory was the most-mentioned theory in response to a question about what theories or research are relied on to inform a plan of how to support a child. At times, this has been cited as evidence of the theory’s popularity (see e.g. Forslund et al. 2022). However, attachment theory was still only mentioned by 11 percent of survey respondents. Furthermore, only approximately 9 percent of respondents were from social care, and the report stated that ‘social workers discussed systemic theory’ (Department for Education 2018: 15). The report did not specify whether this was in addition to, or instead of, attachment theory. This survey does not therefore support arguments for ‘the ubiquity of attachment theory’ (Smith, Cameron, and Reimer 2017: 1067).

In survey research with fifty-five child protection practitioners in Australia, Menzies and Grace (2022) found that attachment was the theory most commonly identified as guiding their practice. According to the survey, 73 percent of respondents stated that they used attachment theory in their day-to-day practice. However, attachment theory was one of three theories given as examples in the survey, and it is unknown if—and to what extent—this may have inflated the response rate. Furthermore, far fewer (33 percent) of respondents stated that they used attachment theory in practice with Aboriginal families. This suggests that the social workers were making decisions to use attachment theory in some contexts and not others, which somewhat counters suggestions it operates as a dominant idea.

In Sweden, Hammarlund et al. (2022) conducted survey research with 191 child protection workers. Hammarlund et al. found that the majority of respondents reported that they ‘form an opinion about a child’s attachment pattern’ in most or all of their child protection investigations. These findings potentially indicate the widespread use of attachment theory amongst these social workers. Similarly, as part of broader research on social work practice in relation to emotional abuse, North (2019) found that social workers in her study almost uniformly stated a view that attachment theory is useful for identifying problematic parent–child relationships. However, as the researchers themselves acknowledge, in both these studies, it is unknown how much emphasis was placed on this information and the extent to which other factors and theoretical perspectives were considered, and thus it is unknown whether attachment theory was a dominant part of the social workers’ thinking.

Studies from several countries (Denmark: Bjerre Madsen, and Petersen 2023; Australia: McLean et al. 2013; Sweden: Alexius and Hollander 2014; UK: Boswell and Cudmore 2017; New Zealand: Keddell 2017) have found social workers making reference to ‘attachment’. The social workers’ references to attachment were not always aligned with attachment theory however. This has also been raised as a point of concern by Forslund et al. (2022). Bjerre Madsen, and Petersen (2023: 60) concluded that their findings indicate that attachment theory has influence as a 'normative cultural form'. This appears to lend support to White et al.’s (2020) argument of attachment theory influencing practice via social workers’ informal theories. Yet, a complicating factor is that the word ‘attachment’ predates attachment theory and continues to exist and have meaning and usage outside of attachment theory. The ordinary language meaning of attachment is very different from the attachment theory meaning of attachment. Some references to ‘attachment’ may not necessarily be associated with and influenced by attachment theory at all. This may contribute to the appearance of attachment theory having greater dominance than it does. The extent to which this may or may not be the case is currently unclear.

A review of the available empirical evidence therefore highlights that claims of attachment theory being the dominant ‘master theory’ in children’s social work are premature. Whilst attachment theory does appear to be cited as one of the more common theoretical influences, research also seems to suggest that many social workers do not draw on attachment theory. Where social workers do draw on attachment theory, it is unclear to what extent the use of attachment theory inhibits other ideas. Furthermore, although the word ‘attachment’ does seem to be a common part of the social work vocabulary, the relationship between such uses and attachment theory is not always clear.

This article seeks to add to the evidence base exploring questions about the prominence of attachment theory in social work practice.

We ask:

1. Is attachment theory dominant in children’s safeguarding social work?
2. Does the use of attachment theory ideas inhibit other ideas?
3. Do ideas from attachment theory influence practice as a lay theory even when not drawn on as a formal theory?
4. Is attachment theory adopted uncritically by children’s safeguarding social workers?

## Methods

This article draws on work carried out as part of a wider study, involving multiple professional groups (Foster 2023). This article solely draws on an analysis of interviews conducted with social workers. The study was approved by the Northumbria University Faculty of Health and Life Sciences Research Ethics Committee and all study participants provided informed written consent.

### Participants

Study participants were twenty-three social workers with experience working in children’s safeguarding from two English local authorities (twelve from one authority, eleven from another). Relevant service directors within the two participating local authorities passed the study information and invitation to all relevant team leaders, who in turn shared the information with social workers within their teams, asking them to contact the researchers if they wished to take part. Nine participating social workers were based in initial assessment teams, eight in longer-term safeguarding teams, and three delivered a support programme to families with children on a child protection or child in need plan. The remaining three were based in fostering, adoption, and child disability teams but all had prior experience working in initial assessment and/or longer-term safeguarding.

Professional experience ranged from one to twenty-two years (average seven years), and age ranged from twenty-five to fifty-eight (average thirty-seven years). The participants were predominantly female (91 percent), which is broadly reflective of the workforce gender split for the profession.

### Procedure and materials

The study was described to prospective participants as being focused on professionals’ perspectives on relationships and their thinking when conducting child and family assessments. No mention was made of attachment or any related terms in the research recruitment materials or process to avoid introducing self-selection bias towards social workers with particular views on attachment theory, and to avoid priming and demand characteristics, as discussed further below. The interviews were carried out face-to-face from October 2017 to May 2018 and involved discussion of family case vignettes followed by additional interview questions.

Vignettes are ‘short hypothetical accounts reflecting real-world situations’ (Tremblay et al. 2022: 1) to which participants are asked to respond. Their use in qualitative research supports the generation of complex data (Wilks 2004) which can be explored in a situational context (Barter and Renold 1999). The vignettes developed for this study were family cases containing child welfare concerns. They were designed to be an analogue to cases social workers receive in their day-to-day practice, and participants were asked to respond to them from their professional perspective. Whilst vignettes can be presented in a range of formats (Tremblay et al. 2022), written narratives were chosen for this study as initial child safeguarding referrals are often received in written form. Serious Case Reviews were used as the basis of the vignettes, to increase their authenticity. The process of developing and refining the vignettes included checks and feedback from multiple social workers. A further check of the validity of the vignettes was made by asking each research participant how familiar the cases felt to them.

Two family case vignettes were developed and discussed with the participants, though only the first was relevant to the research questions in this article and is considered here. This vignette (see Supplementary material) was designed to contain sufficient welfare concerns with relevance to the attachment that drawing on attachment theory would be meaningful to help understand it, whilst not being designed such that it could only be understood by drawing on attachment theory. The Serious Case Review used as the basis for the vignette (Trench and Griffiths 2014) identified how a focus on neglect in relation to poor home conditions and/or a focus on a label such as attention deficit hyperactivity disorder could detract attention away from issues in parent–child relationships. Semi-structured questions were asked to elicit a detailed discussion of the vignette. Questions were asked about the practitioners’ perception of the level of risk and what they saw as the key features of the case, then about possible reasons for the behaviour of different family members, and finally about intervention and outcomes. No mention was made of ‘attachment’ in the vignette or in the questions asked to support the discussion of the vignette (or during recruitment to the study), allowing for exploration of whether participants drew on ideas about attachment to make sense of a family case when there was no explicit prompt or demand characteristic to do so.

After discussing the family case vignette, additional semi-structured interview questions were asked. These explored the social workers’ perceptions of what was informing their thinking about the family case, followed by their views on formal social work theories in general and their potential role in their day-to-day practice thinking, then narrowing to their views on attachment theory and its potential role in their day-to-day practice thinking. Ordering the questions in this way reduced the potential for priming or demand effects to skew the emphasis participants placed on attachment theory.

The use of vignettes increased the possibility of observing ‘theory-in-use’ (theory use that can be inferred from action) rather than solely ‘espoused theory’ (theory that an individual claims to use) (Argyris, Putnam, and Smith 1985; Osmond, Scott, and Clark 2008). By asking participants to think about and discuss their response to a family case vignette, it was possible to observe whether and how ideas from attachment theory informed participants’ thinking even where participants did not explicitly reference the theory and/or view themselves as drawing on the theory. It was also possible to examine whether the family case was thought about differently if attachment theory was not drawn on. Furthermore, combining discussion of a family case vignette with direct follow-on questions about what participants saw themselves as drawing on and their views on and use of attachment theory meant it was possible to see whether there was any misalignment between participants’ espoused theory and theory-in-use.

## Findings

### Espoused views on whether formal theories, including attachment theory, are drawn on in practice

When reflecting on what they drew on to make sense of the family case vignette, most social workers highlighted the central role of their practice experience. Mention of use of any theory was much less common.

For nearly half of the social workers in the sample, formal theories were not something they thought played a role in their assessment of the case or in their day-to-day social work practice. A few described feeling quite daunted by theory: with it seen as something they ‘struggle with’ (Lily) and are ‘rubbish at’ (Poppy). Some described theory as something that had faded from view over time in practice. For example, Holly said, ‘when I started … you go back to the theory that you’ve learnt at university, but the more you do it you rely more on your experience of working with families’. A view of theory as something separate from the primary social work task was also revealed by Primrose’s statement that ‘in practice we don’t use that much theory, and as social workers visiting the family we’re focusing on the task in hand’.

For another group of social workers there was a sense that they viewed theory as something that had the potential to be helpful but thought that reading or thinking about theory after qualification was challenging in the face of the demands of professional practice. Violet said, ‘when I have space and the time … you can bring in theory. But I think on a day-to-day level there isn’t really the space to do that’. Similarly, Rowan said he thought theory could be helpful but ‘thinking about cases theoretically is, sometimes there isn’t time for that’. The idea that there is insufficient time to draw on theory suggests that these social workers viewed theory as something that would need adding on to what they are already doing, rather than as something that could be embedded and subsumed into their thinking and practice.

In contrast, for a third group of social workers, theory was viewed as subsumed into their thinking and practice. There was variation in the extent to which these social workers could trace theoretical ideas back to source and the extent to which they chose to make explicit references to theory, however. At one end of the spectrum, Heather was aware which theories were informing her thinking but chose not to refer to them explicitly in her day-to-day practice: ‘There’s some theory … there’s a few different ones that I would be thinking of, but … I wouldn’t say it, I would only be thinking it’. For others, drawing on theory was described as something they are not always aware of at the time, but can bring awareness to. For example, Jasmine said, ‘I think you draw on elements of lots of things, and sometimes it’s not until you sit and reflect afterwards … that you recognize that’. At the other end of the spectrum were social workers who believed their thinking aligned with theory they had been taught during qualification but were not sure which. For example, Camellia said, ‘I’m sure it would fit in some box somewhere but it’s so long since I’ve been a student … those days are gone, it just comes instinctively now’.

For those who drew on formal theories in their social work practice, some had fully or partially turned away from attachment theory: drawing only on other theories, or drawing predominantly on other theories but with some limited use of attachment theory. Other social workers described attachment theory as being one of several theories in their ‘toolbox’. Systems theory was identified as one of the other theories drawn on by almost all the social workers who used theory. There was just one social worker in this sample who stated that they drew on attachment theory but no other formal theories.

### The extent to which ideas about attachment informed thinking about a family case

In over half the interviews, attachment was not explicitly mentioned by the social workers in their responses to the family case vignette. For many of these social workers, implicit use of attachment theory ideas was not observed in their discussion of the case either, nor did these social workers later identify attachment theory as something they had been drawing on to support their thinking about the case.

Where there was mention of the word attachment during the discussion of the family case, this did not always mean attachment theory was being drawn on. In some interviews, participants used the word attachment as a synonym for the parent–child relationship as a whole or the general emotional bond from child to parent or parent to child. For example, Zinnia said about the mother in the case, ‘if she’s got postnatal depression now, did she have it when the kids … were younger and she’s never formed an attachment to them?’ These references were not to the concept of attachment as defined by attachment theory, or to ideas within attachment theory, but rather to everyday meanings of the word attachment. However, choice of the word attachment, as opposed to cognates, may come partly from its salience in the context of the standing of attachment theory within social work theory.

Some social workers were observed drawing on attachment theory ideas during discussion of the family case, though there was great variation in the depth of engagement with the theory. The social workers who drew on attachment theory when responding to the case typically did so alongside other theories.

Where social workers did not draw on attachment theory ideas explicitly or implicitly, there was often a dominance of social learning explanations, with behaviour understood as likely copied or mimicked. For example, when talking about why the boys in the case might be behaving violently, Willow said, ‘they must have seen that somewhere to behave in that way’ and Zinnia speculated, ‘it could be just mirrored behaviour’. Lack of stimulation, lack of boundaries, and attention-seeking were other common possible explanations proposed for the children’s behaviour in the case vignette. Correspondingly, parenting was discussed in terms of providing stimulation, setting boundaries, providing advice and guidance, and meeting physical needs. Such factors were often considered alongside emotional factors by those who drew on attachment theory ideas to inform their thinking. Yet, for some of those who did not draw explicitly or implicitly on attachment theory, children’s and parents’ behaviour was solely considered in practical and non-emotional terms.

Intergenerational cycles were often mentioned by social workers when thinking about the behaviour of the parents in the case. However, the focus was typically on how parents learn parenting practices and norms through observing and replicating the parenting practices they were exposed to in their childhood. For example, Primrose discussed how ‘it’s like a vicious cycle … it’s copy-cat behaviour from when they were children, and they may see that it’s the norm of parenting’. Therefore, whilst intergenerational cycles are a phenomenon that attachment theory and research have considered, the majority of the social workers in the sample were not observed to be influenced explicitly or implicitly by attachment theory ideas and/or attachment research findings when thinking about intergenerational patterns.

### Critical thinking about attachment theory

There were multiple examples in the sample of social workers taking a critical stance. Some social workers focused on perceived flaws in the theory as a whole. For example, Willow reported turning away from attachment theory because of a perception that the theory was outdated, mother-blaming and not culturally inclusive:

I did do a bit more reading about it [attachment theory] and then found out feminist critiques of it, and …when it was devised and the context at that time, and how things have changed, and then it doesn’t, culturally there’s differences, so … I didn’t like it. … I felt like it blamed mothers for things going wrong.

Some social workers were observed thinking critically about particular aspects of attachment theory and how those aspects might be used in social work practice. Multiple social workers in the sample highlighted potential issues with the use of attachment classification terms in child welfare practice. Concerns were raised that the use of attachment classification terms in child welfare practice can be pathologizing, reductionistic, over-encompassing, and unintelligible for families. For these social workers, there was evidence that these critical insights were informing the ways they did and did not use the theory in practice. For example, Fern said:

I try not to put labels on, this child is suffering disorganized or ambivalent attachment. … It’s focusing more on the behaviours and what the behaviours will be telling you as opposed to sticking a label, an attachment theory label on it. Because it’s just words, we need to understand what that means for these children and what we’re actually seeing.

Social workers in the sample were also observed to be reflecting critically on some of the ways practitioners use attachment theory in their day-to-day practice. For example, Ash said:

I think it can be vague and I think it can be overused by people, or not always used appropriately. So I sometimes read reports or I hear professionals talking about bad attachment and good attachment, and … I don’t think it really explains anything.

Social workers were also observed thinking critically about the extent to which attachment theory should be drawn on in practice. For example, Azalea said ‘attachment theory is a fantastic theory, but actually over-relying on it can be more harmful for children’. A view of the theory being valuable only if used as part of a holistic approach was also expressed by Fern:

I think it’s helpful in terms of something to think about and something to consider. … I don’t think it’s the be all and end all. I think it’s one aspect of everything that you take into consideration and you look at when you’re assessing.

## Discussion

Multiple commentators have claimed that attachment theory is a dominant perspective in social work practice (e.g., Smith, Cameron, and Reimer 2017; White et al. 2020; Garrett 2023; Steggall and Scollen 2024). In the current study, we sought to empirically examine this claim in a sample of children’s safeguarding social workers from two English local authorities. We found widespread awareness of attachment theory but did not find that this translated into attachment theory being a dominant perspective. Some social workers had an anti-theoretical orientation, viewing formal theories in general as having limited value for their day-to-day practice. As a result, these social workers did not tend to explicitly draw on any formal theories including (but not limited to) attachment theory. Hostility towards any academically-grounded theories in UK social work has been found in some previous research (Hicks 2016).

Other social workers saw value in the use of formal theory in their practice, but this was not always associated with the use of attachment theory. In this sample, systems theory was a more commonly cited theoretical influence, with attachment theory—where drawn on—typically used alongside systems theory. This finding appears to run counter to Smith, Cameron, and Reimer's (2017) argument that attachment is the ‘master theory’ informing social work and obstructing consideration of other ideas.

Some previous commentators have argued that attachment theory ideas have permeated into cultural discourses and therefore can influence social workers’ informal theories and practice even if the social workers do not deliberately and consciously draw on attachment theory in their practice (White et al. 2020; Garrett 2023). A benefit of including a family case vignette discussion in the current study was that it was possible to observe if attachment theory ideas were or were not influencing practice thinking in this way. We found that, for some social workers, their understanding of the family case vignette was not influenced by or aligned with an attachment theory perspective, thus countering previous suggestions that attachment theory ideas are pervasive. An alternative discourse used to explain children’s behaviour was to regard it as observed and copied. Social workers drawing on this alternative discourse were not deliberately and consciously drawing on social learning theory (Bandura 1977), but ideas aligned with social learning theory appeared to be influencing their informal theories and practice understanding. We did therefore find that formal theoretical ideas could influence social workers’ informal theories, but attachment theory did not have dominance, or even an especially competitive position, as such an influence.

A further theme across previous commentaries on the use of attachment theory in social work practice has been that social workers are insufficiently critical: either about the theory itself (Garrett 2023) or about whether it is being applied to practice in appropriate ways (White et al. 2020). In contrast with this depiction, we found evidence that some social workers are engaging critically with attachment theory and its practice applications.

Some social workers had read polemical critiques of attachment theory that take issue with the validity of attachment theory as a whole whilst solely focusing on early work by Bowlby and Ainsworth. Garrett (2023) is one illustration of such critique, another is Vicedo (2017). Such criticisms ignore the decades of work on attachment after Bowlby and Ainsworth, and even where these scholars changed their minds later in their careers (Duschinsky et al. 2020). However, where social workers only knew of the early work by Bowlby and Ainsworth, these critiques could be successful in convincing social workers that attachment theory was weak and unhelpful.

Other social workers were found to be thinking critically about the implications of different practice uses of attachment theory. Much of this critical thinking centred around attachment classifications. In clear contrast to White et al.’s (2020: viii) argument that the classifications provide social workers with ‘a degree of comfort’, ‘a handy vocabulary’ and are widely and enthusiastically adopted (see also Smith, Cameron, and Reimer 2017), most social workers in the current study were either ambivalent or very critical about practice use of attachment classifications.

### Strengths and limitations of the study

Having been critical of the limited evidence base for some previous claims about the dominance of attachment theory in practice, it is important to be clear about the strengths and limitations of this study and the implications for what we can and cannot be claimed. As an exploratory qualitative study, this study was not designed to recruit a necessarily representative sample and produce statistically generalizable findings (Smith 2018). Whilst we found that attachment theory was not a dominant perspective in this sample, we cannot conclude from this that it is not the dominant perspective for some other social workers or social work teams. However, just as the discovery of one black swan is sufficient to counter claims that all swans are white, the finding from this research that numerous children’s safeguarding social workers do not explicitly or implicitly draw on attachment theory is sufficient to highlight that claims about the dominance of attachment theory in children’s safeguarding social work practice should not be accepted unquestioningly.

Furthermore, whilst statistical generalizability is not a relevant concept for judging the value of qualitative research, the concept of transferability—the extent to which findings are transferable to other settings—is a relevant consideration (Smith 2018). Several factors provide confidence that the findings have transferability beyond the twenty-three social workers involved in the research. First, the participants were from two local authorities, meaning the sample was not at risk of solely representing practices relating to a particular context within a single organization (unlike in Gibson’s 2016 research). Secondly, the participants were from multiple teams within the two local authorities, meaning the sample was not at risk of solely representing an unusual standpoint developed by a particular manager and permeated through a team. Thirdly, the social workers had undertaken their pre-qualifying education via several providers and at a range of times and had not all undertaken further attachment-related training. Thus, the sample was not at risk of solely representing a specific standpoint encouraged by particular training. Fourthly, recruitment did not make any mention of ‘attachment’, so the sample was not at risk of self-selection bias towards, and thus over-representing, those who particularly value attachment theory. It is important to note, however, that this research focused only on children’s safeguarding social work and does not provide any insights into the prominence of attachment theory in fostering and adoption social work.

While the recruitment process for the study deliberately avoided mention of attachment theory, or indeed any theory, the study was clearly identified as research being conducted by academics. There is the potential for this to have led to some self-selection bias towards social workers who particularly value academic theories and/or for participants to reference theory more than they do in day-to-day practice. If this was the case, then the substantial proportion of participants who did not mention or draw on theory and professed themselves to not use theory is arguably even more noteworthy. It is also possible that there was some self-selection bias towards social workers who are more reflective and critical about influences on their practice thinking, including attachment theory. Making time to participate in this research could have been more attractive to such social workers. This does not undermine the ability to conclude from this study that there are social workers who engage with attachment theory thoughtfully and critically, but does reinforce the earlier point cautioning against conclusions that the proportion who were critical in this sample will necessarily equate to the proportion who are critical in the wider social worker population.

Use of the vignette method allowed for direct observation of how practitioners drew on (or did not draw on) attachment theory ideas in their thinking about a family case, rather than just their reported use of attachment theory ideas. This was particularly valuable in light of previous findings that social workers who think they are drawing on attachment theory are sometimes drawing on lay understandings of attachment instead (e.g. Bjerre Madsen, and Petersen 2023), and the argument that social workers who do not think they are drawing on attachment theory may sometimes be doing so indirectly if the theory has influenced their informal theories (e.g. White et al. 2020). The use of a vignette also enabled insights into thought processes that are likely to remain out of view in research that observes direct practice or examines child protection assessment records (e.g. Gibson 2016). Whilst these are valuable strengths of the vignette method, it is also important to acknowledge that vignettes provide an analogue to practice rather than direct access to practice.

### Implications for practice

The family case vignette used in this research study contained a wide range of potentially concerning factors and much was uncertain or unknown, as is often the case in practice. As a result, while it was not designed to have a ‘right answer’, perspectives on the case that overlooked its complexity and uncertainty could be considered less ideal. There are contrasting views in the literature on whether the use of an attachment theory perspective is likely to reduce or exacerbate the overlooking of factors. In this study, attachment theory tended to only be drawn on by social workers as part of a wider set of considerations. The concerns that some previous commentators (e.g. Smith, Cameron, and Reimer 2017) have directed at attachment theory—that its use in practice inhibits other ideas—were not seen. Indeed, examples of leaning on singular explanations when discussing aspects of the case were more commonly observed among the social workers who did not draw on attachment theory. This study therefore lends further support to recommendations (such as those from Brandon et al. 2011 and Munro 2011) that practitioners should understand attachment theory and be curious about attachment and parent–child relationships, and that this has the potential to reduce the overlooking of some factors rather than exacerbate the overlooking of other factors. Furthermore, the limited use of formal theories in general, including attachment theory, in this sample suggests that more could be done to support social workers to consider how theory could enhance and be embedded into their day-to-day practice.

## Conclusion

In this article, we have drawn attention to critical arguments in circulation about the prominence of attachment theory in practice. We agree that it would be concerning if a single theory is informing social work practice in a dominant, pervasive, and uncritically accepted way. Yet, these concerns are not a response to robust empirical evidence of the place of attachment theory in social work practice.

The current study findings challenge depictions of attachment theory as a prominent, often dominant, perspective in children’s safeguarding social work practice. The findings also challenge depictions of social workers as being insufficiently critical of attachment theory and its practice applications. It would be premature on the basis of one qualitative study to conclude that attachment theory is not a dominant perspective anywhere in social work practice, but what this study does highlight is that we should not uncritically accept claims that attachment theory is dominant either.

Smith, Cameron, and Reimer (2017: 1618) stated that their aim was to ‘put a stutter into the seemingly inexorable turn to attachment theory in children and families social work’. If attachment theory has the dominance that Smith et al. portray, this may be a laudable aim, and helpful in encouraging social workers to balance the use of an attachment theory perspective with other perspectives. Yet, if attachment theory actually has much less prominence, as our empirical research suggests it may, such arguments could instead encourage social workers to reduce the use of attachment theory to the point that they may overlook some factors of importance when working with families (Brandon et al. 2011).

We therefore encourage care and nuance from those making arguments about what might be the appropriate level of reliance on attachment theory in social work practice. Discussions on whether the right emphasis is given to attachment theory in practice would benefit from being grounded in more robust research on the current level of emphasis. Research looking to ascertain how influential attachment theory is should be careful to delineate between the use of attachment theory, the use of the term attachment due to the apparent standing of the theory but without substantively taking anything from the theory, and the use of the word attachment with no attempted link to attachment theory. Without this, arguments can start to take the form of moral panic and could encourage a turning away from all attachment theory and research. This could result in missed opportunities to use relevant insights from the theory to enhance work with children and families.

## Supplementary Material

bcaf068_Supplementary_Data
