# Supplementary material for: ‘In practice we don’t use that much theory’: Questioning claims of the dominance of attachment theory in children’s safeguarding social work
Source: Br J Soc Work. 2025 Mar 28;55(6):2716–33. doi: 10.1093/bjsw/bcaf068 (PMC12465119; doi:10.1093/bjsw/bcaf068)
Supplement: bcaf068_Supplementary_Data [file bcaf068_supplementary_data.docx]

**Supplementary material: Case vignette**

Mother – Jade, aged 35

Father – Alex, aged 37

Son – Sam, aged 8

Son – Tom, aged 6

Daughter – Poppy, aged 6 months

Jade was known to the locality team as a child. She was on a child protection plan for sixteen months from the age of two and placed in foster care on a care order from the age of five to seven. Jade has ongoing depression and anxiety, and is taking anti-depressant medication.

Over the past five years there have been a number of reported incidents of domestic abuse. These include reports from Jade herself about Alex’s violence, and others from neighbours when they heard angry scenes in the home. Alex no longer lives with the family, but sees them on a regular basis and is sometimes the carer for the children.

During Jade’s recent pregnancy, the midwife asked about the domestic abuse. Jade said she ‘didn’t want to discuss it’. She said that the threat from Alex and their upsets and arguments were ‘all in the past’, and that there was no current violence.

There have been several reported accidents involving the children, which appear to relate to inadequate supervision. Since the birth of Poppy, there have also been a number of missed essential health appointments for the children. The Health Visitor’s records note that Jade ‘has difficulty providing stimulation for the children and often leaves them in front of the TV’. The Health Visitor has also noted the poor state of the family home: unclean and very cluttered, with not enough space for the baby to develop physically. The Health Visitor believes Jade ‘has good intentions, but easily forgets.’

Jade says that she is unable to manage her children’s behaviour. Sam has been diagnosed with ADHD and receives medication for this. Jade has suggested to professionals that Tom also has ADHD, reporting that he shows ‘wild behaviour’, but clinical assessment indicated that he did not meet diagnostic criteria for ADHD. Jade has asked for an assessment of Autism Spectrum Disorder for Tom. Jade describes Tom as violent and out-of-control at home and says that he and Sam fight and risk physically hurting each other.

The children regularly arrive late at school and there is a high, and increasing, level of school absences. Teachers have noted that the boys’ academic progress is below average in all areas of the curriculum. Tom is described by his teacher as quite quiet and subdued at school. The SENCO has expressed concerns about Tom’s ‘extremely withdrawn and unhappy behaviour’ at school and made a referral.
